# The associations between organizational culture, organizational structure and quality management in European hospitals

**DOI:** 10.1093/intqhc/mzu027

**Published:** 2014-03-25

**Authors:** C. Wagner, R. Mannion, A. Hammer, O. Groene, O.A. Arah, M. Dersarkissian, R. Suñol, on behalf of the DUQuE Project Consortium

**Affiliations:** 1 NIVEL Netherlands Institute for Health Services Research, Utrecht, The Netherlands; 2 Department of Public and Occupational Health, EMGO Institute for Health and Care Research, VU University Medical Center, Amsterdam, The Netherlands; 3 Health Services Management Centre, University of Birmingham, Birmingham B15 2RT, UK; 4 Faculty of Human Science and Faculty of Medicine, Institute for Medical Sociology, Health Services Research and Rehabilitation Science, University of Cologne, Cologne, Germany; 5 Department of Health Services Research and Policy, London School of Hygiene and Tropical Medicine (LSHTM), London, UK; 6 Department of Epidemiology, Fielding School of Public Health, University of California, Los Angeles (UCLA), Los Angeles, CA, USA; 7 UCLA Center for Health Policy Research, Los Angeles, CA, USA; 8 Avedis Donabedian Research Institute (FAD), Universitat Autonoma de Barcelona, Barcelona, Spain; 9 Red de Investigación en Servicios de Salud en Enfermedades Crónicas (REDISSEC), Barcelona, Spain

**Keywords:** organizational culture, organizational structure, hospital, quality management, quality improvement, DUQuE

## Abstract

**Objective:**

To better understand associations between organizational culture (OC), organizational management structure (OS) and quality management in hospitals.

**Design:**

A multi-method, multi-level, cross-sectional observational study.

**Setting and participants:**

As part of the DUQuE project (Deepening our Understanding of Quality improvement in Europe), a random sample of 188 hospitals in 7 countries (France, Poland, Turkey, Portugal, Spain, Germany and Czech Republic) participated in a comprehensive questionnaire survey and a one-day on-site surveyor audit. Respondents for this study (*n* = 158) included professional quality managers and hospital trustees.

**Main outcome measures:**

Extent of implementation of quality management systems, extent of compliance with existing management procedures and implementation of clinical quality activities.

**Results:**

Among participating hospitals, 33% had a clan culture as their dominant culture type, 26% an open and developmental culture type, 16% a hierarchical culture type and 25% a rational culture type. The culture type had no statistically significant association with the outcome measures. Some structural characteristics were associated with the development of quality management systems.

**Conclusion:**

The type of OC was not associated with the development of quality management in hospitals. Other factors (not culture type) are associated with the development of quality management. An OS that uses fewer protocols is associated with a less developed quality management system, whereas an OS which supports innovation in care is associated with a more developed quality management system.

## Introduction

Many cultural aspects of health-care organizations are understood to be important in determining the quality of patient care—whether through fostering excellence or contributing to failure [1–4]. Organizational culture (OC) represents the shared beliefs, values, attitudes, norms of behaviour of people in an organization and the established organizational routines, traditions, ceremonies and reward systems. OC defines legitimate and acceptable actions within an organization and encompasses the meanings that professionals and staff assign to their work. It is the social and normative ‘glue’ that binds people into collective enterprise; and it defines ‘the way things are done around here’.

OC s vary. There is international interest in using OC as a lever for health system reform. Previous research has found that a participative, flexible, risk-taking ‘developmental’ OC was significantly related to the implementation of quality improvement activities in hospitals in the USA [5]. However, few studies have explored the relationships among OC, organizational management structure (OS) and implementation of quality improvement activities.

In this paper we explore the role of OC and OS as influences on the implementation of quality improvement in European hospitals. In particular, we investigate the interaction between types of OC and OS s and assess the impact of this interaction on quality management strategies. We test the hypothesis that more quality management strategies will be implemented in hospitals that demonstrate congruence between the dominant OC and the perceived OS (and conversely, that hospitals with a mismatch between culture and structure will have less implementation of quality management strategies. The central research questions are as follows:

1. Is there a relation between OC and quality management strategies at the hospital level, and between OS and quality management strategies?
2. Is there an interaction between OC and structure that influences the implementation of quality management strategies?

## Methods

### Conceptual framework

**Figure 1:**
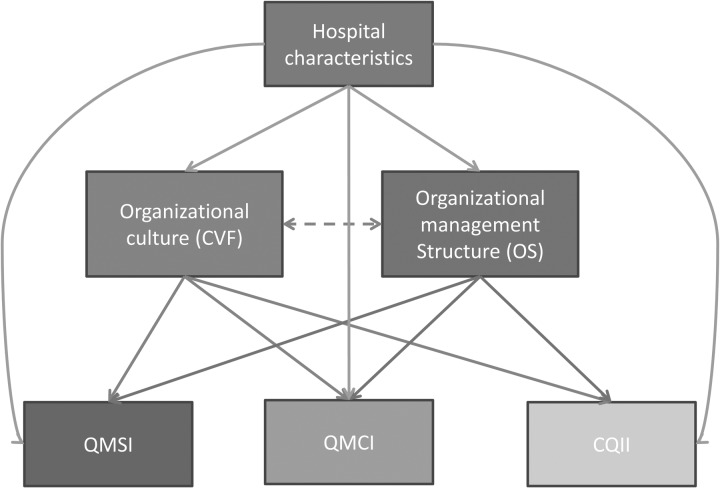
DAG showing the relationships between the study variables. QMSI, Quality Management System Index; QMCI, Quality Management Compliance Index; CQII, Clinical Quality Implementation Index.

The purpose of this paper is to test the relationship between OC, OS (OS) and quality management initiatives, measured in a large sample of hospital units by calculating three indices, e.g. Quality Management System Index (QMSI), Quality Management Compliance Index (QMCI) and Clinical Quality Implementation Index (CQII). To guide covariate selection for confounding control in our analysis, we constructed the directed acyclic graph (DAG), shown in Fig. 1. The DAG represents the underlying data generating mechanism of interest and shows relationships between variables in our analysis [6].

### Setting, study design and population

This analysis was part of a larger project, ‘Deepening our Understanding of Quality improvement in Europe’ (DUQuE) supported by the European Union [7]. We approached 210 hospitals selected at random in Czech Republic, France, Germany, Poland, Portugal, Spain and Turkey; 188 agreed to participate (each with a minimum of 130 beds). Four clinical services (care for acute myocardial infection, stroke, hip fracture and obstetrical services) were chosen because of their relatively high costs, high prevalence of the presenting condition and involvement of distinct types of patients and clinical specialists. Data were collected at hospital, departmental, professional and patient levels using a multi-method approach involving audits and several professional questionnaires to evaluate the maturity of hospital quality management systems and the effects of these systems on organizational, departmental and patient-related quality of care. Ethical approval was gained by the project coordinator at the Bioethics Committee of the Health Department of the Government of Catalonia (Spain). As required, the study was also approved by national ethical committees in each of the states. Additional detailed information about study setting, population and design can be found elsewhere [8].

For the purpose of the analysis, we used five modules of questionnaire and audit-based measures of the hospital capturing OC, OS, QMSI, QMCI and CQII). The first two modules were modelled as predictors, while the latter three were the study outcomes of interest.

### Measuring OC using the competing values framework

Hospital OC was measured using the competing values framework (CVF) [3]. The CVF uses two main dimensions—the first describing how internal processes are structured within the hospital and the second describing the orientation of the hospital to the external world. This gives rise to four distinct organizational cultural ‘types’, e.g. clan culture, developmental culture (also known as open culture), hierarchical culture and rational culture (see Box 1). Crucially, hospitals are not deemed to be simply one or other of these four types: instead, each hospital has competing values that involve all four types while nonetheless having a tendency towards one of the quadrants.

**Box 1:**
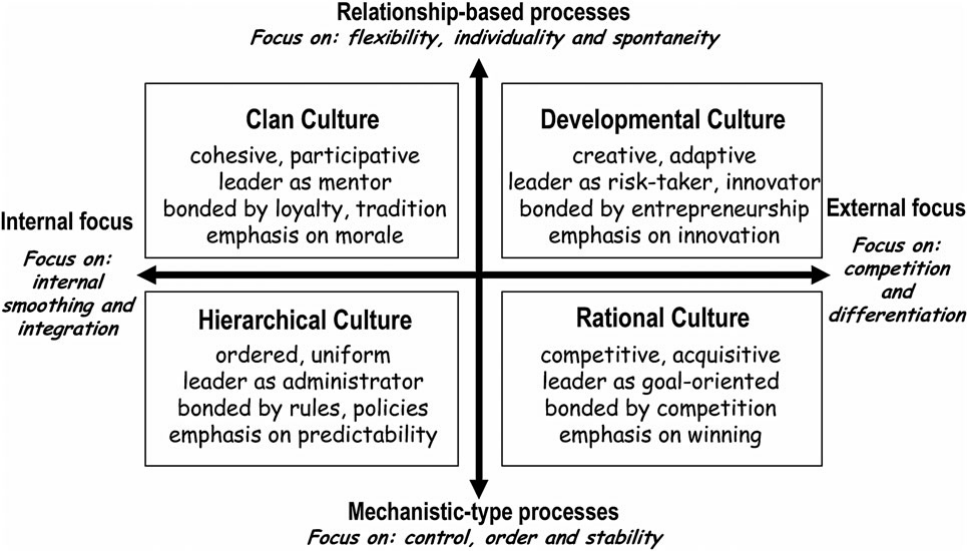
‘The Competing Values Framework’ for modelling organisational culture. See ref. [4].

Measurement of the dominant culture type for a hospital was accomplished through use of the CVF questionnaire which offers respondents a series of descriptions of a hospital, arranged in five aspects of OC with four answer categories representing different culture types. Within each group of four descriptions, the respondent is asked to ‘distribute 100 points’ between them ‘according to which description best fits your current organization’. The five groups represent descriptions of hospital characteristics, leadership, emphasis, cohesion and rewards. Collating these ‘points allocations’ provides a score (in the range of 0–100) for each individual on each of four cultural types. The CVF is considered valid with three or more respondents so that the CVF items were incorporated into the questionnaires of the four top-level managers (a board trustee, chief executive officer, the chief medical officer and the highest ranking nurse) [4]. Hospital culture types were defined based on the average of three respondents with the trustee response used only if a response was missing from one of the others. The dominant culture type for each hospital was based on the highest average culture type score.

### Measuring OS

OS was measured along several dimensions based on responses to a survey completed by the quality manager. On a five-point scale, we measured several structural characteristics of the hospital's management, such as management structure (hierarchical = 1 vs. horizontal = 5); decision making (centralized = 1 vs. decentralized = 5); activities are regulated by protocols (many = 1 vs. few = 5); innovation of care processes (not valued = 1 vs. highly valued = 5). The scores of the individual items were combined to an average score on quality management structure.

### First-study outcome: hospital QMSI

The QMSI is a newly developed multi-item and multi-dimensional instrument measuring the degree of implementation of quality management systems in hospitals [9]. The implementation of the quality management system was determined by the extent to which quality policy documents, internal quality methods, training of professionals, formal protocols, evaluation of results and monitoring quality were implemented in the hospital. The index is based on 46 items (each with a 4-point Likert response scale) that form 9 sub-scales. The instrument was included in the questionnaire for quality managers. For this study, we used a composite score averaged across the nine dimensions. The total QMSI score ranges from 0 to 27 points. Psychometric analyses found satisfactory Cronbach's alpha (ranging from 0.72 to 0.82) for eight of the nine scales and a low Cronbach's alpha (0.48) for the scale ‘analysing feedback and patient experiences’. Because of the theoretical importance this scale was retained. Tests of construct validity found a relationship between the index and performance on recent measures of quality. These results are available from the authors on request.

### Second-study outcome: QMCI

The aim of the QMCI is to identify and externally verify the compliance of a set of interacting activities on four domains, including quality planning, monitoring of patient and professional opinions, and monitoring of the quality system. The total number of activities reviewed by the auditor is 15. During a 1-day visit to each hospital, trained auditors scored these activities on a five-point-likert scale (range 0–4) from ‘No or negligible compliance’ to ‘Full compliance’. The index was constructed by taking the sum of four sub-scales and ranges from 0 to 16. Psychometric analysis revealed a reliable and valid index [10].

### Third-study outcome: CQII

The CQII has been designed to measure to what extent some key quality areas are implemented across the hospital. These areas are preventing hospital infections, medication management, preventing patient falls, preventing patient ulcers, routine testing of elective surgery patients, safe surgery practices and preventing deterioration. During the 1-day visit, trained surveyors scored implementation of each clinical activities on a five-point-Likert scale (range 0–4) from ‘No or negligible compliance’ to ‘Full compliance’. The index was constructed by taking the sum of sub-scales that captured each of the seven key areas. After combining response categories to create a three-point scale by recoding 0 and 1 on the original scale to a 1, recoding 2 and 3 on the original scale to a 2, and recoding 4 on the original scale to a 3) the index score ranged from 0 to 14. Psychometric analysis revealed a valid index [10].

### Data collection

For the purpose of the analysis, we relied on hospital-level questionnaires and 1-day surveyor audit performed at the hospital level. Per hospital, surveys included the chair of the board of trustees, chief executive officers, chief medical officers, highest ranking nurse and quality managers (*n* = 4). We used the mean score of at least three answers. Audits were conducted by trained surveyors who talked with several professionals on site about the items described above for the measures QMCI and CQII. More details about the items are published elsewhere [10]. Data were gathered between May 2011 and February 2012 via web-based questionnaires. All participants were sent passwords to access the web-based questionnaires, and sent two reminders if necessary. In order to protect the anonymity of participants, personal identifiers were erased from the database following the data collection period.

### Hospital characteristics

Hospital characteristics included hospital ownership, teaching status and the number of beds and these data were extracted from country-level databases. Hospitals ownership was classified as public (1) or non-public (0). Hospital teaching status was categorized as general (0) or teaching (1). Hospitals were classified into four categories based on the number of beds (< 200 beds, 200–500 beds, 501–1000 beds and >1000 beds).

### Statistical analyses

We calculated descriptive statistics for the hospitals included in this study and for main predictors, outcomes and covariates. For categorical variables we calculated frequencies and percentages. For continuous variables, we calculated the mean, standard deviation and range. For the multivariable-adjusted analysis aimed at our core research objective, we ran separate multi-level linear regression models for each outcome, basing our selection of variables used to control for potential confounding on the DAG (Fig. 1). We ran two models for each outcome under study. In the first model, we fit a multivariate linear mixed model regression with random intercept (by country), adjusted for hospital size, teaching status, ownership and hospital organizational structure. In the second model, we added interaction terms between hospital OC types and hospital organizational structure. For both models we calculated regression coefficients, standard error and *P*-values. Since the data were collected in seven countries we used multi-level modelling to account for clustering of hospitals within countries. We conducted all analyses using Statistical Analysis Software (SAS) version 9.3 (SAS Institute, Inc., Cary, NC, USA, 2012).

## Results

Characteristics of the participating hospitals are summarized in Table 1. Overall, 188 out of 210 hospitals (response rate = 89.5%) participated in the DUQuE study. The final data set used for these analyses contains 158 hospitals for the analysis with the QMSI and 64 hospitals for analysis with QMCI and CQII. Most hospitals were public hospitals (81%), nearly half had teaching status (42%) and 44% were medium sized (between 200 and 1000 beds).

**Table 1:** Characteristics of hospitals used in analysis (*n* = 158)

Characteristic	*n* (%)
Hospitals used in analysis	158 (100)
Czech Republic	26 (16.4)
France	20 (12.6)
Germany	11 (6.9)
Poland	26 (16.4)
Portugal	25 (15.8)
Spain	26 (16.4)
Turkey	24 (15.1)
Teaching hospitals	67 (42.4)
Public hospitals	128 (81.0)
Approximate number of beds in hospital	
<200	16 (10.1)
200–500	69 (43.6)
501–1000	51 (32.2)
>1000	22 (13.9)

Table 2 summarizes the organizational types and structures. One-third of the hospitals have a clan culture as their dominant culture type (meaning a focus on staff empowerment, team building, employee involvement and open communication as approaches to quality improvement). One-quarter have an open, developmental culture (a focus on creative and innovative approaches and solutions to quality improvement, underpinned by the belief that innovative and pioneering initiatives lead to success). One quarter have a predominantly rational culture type (emphasis on measuring consumer/patient preferences, improving competition and market share because consumers are perceived to be selective and efficiency and productivity are key drivers). Hierarchy as the dominant culture type was the least common (16%). Hierarchical cultures are characterized as having clear lines of decision-making authority, formalized measurement and error detection systems, top-down monitoring and process control, and formal rules and policies. Some culture types appeared more prevalent in some countries compared with others.

**Table 2:** Descriptive statistics of OC, quality management indices and OS (*n* = 158)

	Mean (SD)
Predictor variables (scale)	
OC, *n* (%)^a^	
Clan	52 (32.9)
Open/developmental	42 (26.5)
Hierarchy	25 (15.8)
Rational	39 (24.6)
Outcome variables (scale)	
QMSI (0–27)	19.4 (4.6)
QMCI (0–16)^b^	10.4 (3.1)
CQII (0–14)^b^	8.4 (2.9)
Covariates	
Organizational structure (1 = hierarchical to 5 = horizontal)	2.0 (1.0)
Type of decision-making (1 = centralized to 5 = decentralized)	2.1 (1.0)
Number of activities regulated by protocols (1 = many to 5 = few)	2.2 (1.1)
Value of innovation of care processes (1 = not valued to 5 = highly valued)	3.4 (1.0)

^a^Number of hospitals assigned to each organizational type (based on dominant type).

^b^64 hospitals are in-depth hospitals in this analysis; QMCI and CQII are only measured in in-depth hospitals.

We found no associations between CVF culture type and the implementation of quality management strategies as measured by the QMSI, QMCI and CQII (Tables 3–5). However, OS was associated with the implementation of QMSI. A one unit change towards a more horizontal organizational structure was associated with higher QMSI scores on average (b = 0.59; *P* = 0.0415). Organizations that placed higher value on innovation in care processes had, on average, a higher score on the QMSI (b = 1.71; *P* < 0.0001). Hospital organizational structures with fewer activities regulated by protocols were associated with a higher score on the QMSI (b = −1.71; *P* = < 0.0001). The OS had no association with the QMCI or the CQII. We found no statistically significant interaction effects between OC type and OS for any of the three quality management measures used as dependent variables.

**Table 3:** Regression coefficient estimates (standard errors) for the associations of hospital-level OC and OS with quality management systems index (QMSI) (*n* = 158)

	Model 1^a^	
	*b* (SE)	*P*-value
OC type	–	–
Rational	1.15 (0.77)	0.1387
Hierarchy	0.44 (0.83)	0.5975
Open/developmental	0.50 (0.77)	0.5117
Clan	(ref.)	
Organizational structure (1 = hierarchical to 5 = horizontal)	0.59 (0.29)	0.0415
Type of decision-making (1 = centralized to 5 = decentralized)	0.16 (0.28)	0.5736
Number of activities regulated by protocols (1 = many to 5 = few)	−1.71 (0.29)	<0.0001
Value of innovation care processes (1 = not valued to 5 = highly valued)	1.71 (0.31)	<0.0001

^a^Multivariate linear mixed model adjusted for fixed effects at the hospital level (number of beds, teaching status and ownership) and organizational structure.

**Table 4:** Regression coefficient estimates (standard errors) for the associations of hospital-level OC and OS with quality compliance index (QMCI) (*n* = 64)

	Model 1^a^	
	*b* (SE)	*P*-value
OC type	–	–
Rational	−0.41 (1.13)	0.7200
Hierarchy	0.75 (1.29)	0.5619
Open/developmental	1.38 (1.12)	0.2272
Clan	(ref.)	
Organizational structure	−0.03 (0.55)	0.9606
Type of decision-making	0.08 (0.51)	0.8690
Number of activities regulated by protocols	−0.88 (0.41)	0.0356
Value of innovation care processes	−0.04 (0.43)	0.9247

^a^Multivariate linear mixed model adjusted for fixed effects at the hospital level (number of beds, teaching status and ownership) and organizational structure.

**Table 5:** Regression coefficient estimates (standard errors) for the associations of hospital-level OC and OS with CQII (*n* = 64)

	Model 1^a^	
	*b* (SE)	*P*-value
OC type	–	–
Rational	−1.94 (1.05)	0.0704
Hierarchy	−1.75 (1.19)	0.147
Open/developmental	−0.25 (1.02)	0.8101
Clan	(ref)	
Organizational structure	−0.19 (0.5)	0.7085
Type of decision-making	0.40 (0.46)	0.3909
Number of activities regulated by protocols	−0.50 (0.39)	0.206
Value of innovation care processes	−0.05 (0.41)	0.9116

^a^Multivariate linear mixed model adjusted for fixed effects at the hospital level (number of beds, teaching status and ownership) and organizational structure.

## Discussion

This is the first large-scale study which has explored the relationship between OCs, structures and quality management in hospitals. In general, our results do not support the notion that quality management strategies are more developed where OCs and structures are aligned. We found that the type of OC in our sample of hospitals was not linked to quality management efforts and that quality improvement activities were undertaken to some extent regardless of culture type. We also found that there was no significant interaction between OC types and OS that appeared to influence the implementation of quality management strategies.

In our study we found that OS is linked to quality management with organizations with fewer activities regulated by protocols having a lower QMSI score. Thus, it would appear that if hospitals wish to improve their performance on the implementation of quality management systems that they need to pay more attention to developing formal protocols, rules and regulations, perhaps attached to incentives, to help enhance activity in this area.

In contrast to our results, an earlier study suggested that a quality management open/developmental culture was positively related to quality management implementation. The study results were based on 61 US hospitals and 7000 respondents [5]. That study was different from ours as the number of respondents was much larger and based on the views of health-care professionals instead of a small number of respondents of the management board of the hospital. Our results suggest there is not one type of culture which is most important for quality management implementation. Congruence between the culture and the structure in each organization may be less important. As organizational structures are probably easier to manipulate than OCs (at least in the short term) attention should focus on diagnosing local cultures and then designing OSs to accommodate these.

Our results should not be misinterpreted as meaning that OC is unimportant in relation to quality management. The values and beliefs that underpin such an activity may shape how quality management programmes are implemented and evaluated. If all hospital culture types are associated with quality management as we observed, that it may be that there is not one best culture to support quality improvement. Hospitals can develop a range of supportive cultures depending on local contexts and contingencies. We did find a positive relationship between OSs and different types of quality management with the implication that hospitals need to develop appropriate OSs to support the types of quality management they are interested in implementing.

Taken together, our findings imply that decision-making for quality improvement should be decentralized to the local level, but this should not be completely permissive. Furthermore, hospitals may require at least some degree of formal guidance to help structure the menu of choices available to support decisions that are made locally.

### Strengths and limitations

The CVF as a measure of OC has a number of advantages and disadvantages. The advantages are that it has a firm theoretical basis, has good face validity and is quick to complete. It has also been validated and used across a wide range of industries, including health care. There are disadvantages also. First, like all models, the CVF measure may be a gross oversimplification of reality. The measure is based on only three responses from the senior management team of each hospital. These executives may have a limited view of OC in their organization [11]. If so, then to capture the rich and dynamic complexities of OC. Secondly, we used different sources of data for dependent and independent variables (questionnaires for different respondents) in order to decrease the risk of common method variance bias [12]. Thirdly, data for this study were collected across seven countries. To account for clustering of hospitals within the countries, we used random intercept by country in our multivariate regression analyses. Finally, to measure QMSI we used quality managers. The use of quality managers is reasonable, because they are presumed to have a comprehensive knowledge about the quality management in their organizations [13].

### Future research

Our study provides several insights for future research. First, it would be beneficial to undertake longitudinal analysis with multiple data points so that inferences about direction of causality can be established. Secondly, future research should attempt to measure OC among frontline staff rather than senior managers. Frontline staff may be better in a better position to describe the culture of their institution, and in particular wards, departments and clinical services. Finally, our study is based on the analysis of quantitative data which may have failed to capture the rich qualitative aspects of organizational life which defy simple quantification and are better researched using in-depth qualitative methods. Future studies might use a mixed methods research designs which combine both quantitative and qualitative approaches to understanding the impact of culture and structure on the implementation of quality improvement in hospital settings.

## Conclusions

It appears that OS does have an impact on the implementation of quality management strategies but that specific OC types are not associated with quality management implementation. There is no single dominant OC type that is more than others positively associated with quality management in European hospitals.

## Funding

Deepening our Understanding of Quality Improvement in Europe (DUQuE) has received funding from the European Community's Seventh Framework Programme (FP7/2007–2013) under grant agreement number 241822.
